# Maize ZmWRKY28: a target to regulate shade avoidance response under high planting density

**DOI:** 10.1093/jxb/erad146

**Published:** 2023-05-19

**Authors:** Nishat S Islam

**Affiliations:** Department of Biology, University of Western Ontario, London, ON, Canada

**Keywords:** Etiolation, maize, photoreceptors, phytochrome-interacting factors (PIFs) skotomorphogenesis, transcription regulation, shade avoidance response (SAR), yield

## Abstract

This article comments on:

**Zhang Z, Chen L, Yu J.** 2023. Maize WRKY28 interacts with the DELLA protein D8 to affect skotomorphogenesis and participates in the regulation of shade avoidance and plant architecture. *Journal of Experimental Botany***74**, 3122–3141.

**To feed the global population sufficiently from a small number of edible crops grown on limited arable lands, dense planting is required. However, high-density planting often induces the shade avoidance response (SAR), which is a limiting factor for yield. Plants undergo vegetative and reproductive changes to adapt to the varying amount and quality of incident light. These complex processes are delicately managed through signal transduction and regulatory systems comprising, but not limited to, receptors and transporters, hormones and specialized metabolites, and transcription factors (TFs). Zhang *et al.* (2023) have identified such a key regulatory element of the maize (*Zea mays*) SAR, ZmWRKY28, and explored its light-depended pathway regulation. They introduced a potential new target for maize yield improvement.**

Based on the availability of light, flowering plants undergo two different developmental programs: skotomorphogenesis, namely growing in darkness, or photomorphogenesis, namely growing in light (Alabadí *et al.*, 2004). The first major event of plant life is germination, which occurs within the soil in darkness. During this phase, the germinating seeds utilize most of the stored materials (mostly carbohydrates and nitrogen compounds) for hypocotyl elongation to reach the light, and to produce photosynthetically active tissues (Leivar *et al.*, 2008). Thus, skotomorphogenic development plays a pivotal role in plant survival. Plants switch from skotomorphogenic to photomorphogenic growth drastically upon receiving the first photons on the soil surface. However, plants can return from photo- to skotomorphogenesis in later stages as well when there is a lack of sufficient light and, by altering their morphology and physiology, they adapt to the change.

CONSTITUTIVE PHOTOMORPHOGENIC 1 (COP1)–LONG HYPOCOTYL 5 (HY5) light signalling regulation is central to light responsiveness in plants (Fernando and Schroeder, 2016; Huai *et al.*, 2020). Skotomorphogenesis is activated by the degradation of HY5 by COP1 and associated proteins. Phytochrome-interacting factors (PIFs), members of the basic helix–loop–helix (bHLH) TF family, work independently or with the COP1–HY5 cycle by receiving signals from the photoreceptor phytochrome (Phy) (Leivar *et al.*, 2008). Light-activated PhyA or PhyB initiates the degradation of PIFs in the nucleus upon phosphorylation, resulting in the de-etiolated phenotypes in plants (Demarsy, 2022; Sheehan *et al.*, 2007). Conversely, the lack of photosynthetically active light inactivates PhyA and PhyB. As a result, PIFs accumulate to induce a series of genes and hormonal signals to respond to the shade. This adaptive response is known as the shade avoidance response (SAR), which causes unexpected tall plant stature and affects the quality and quantity of the grains (Lorrain *et al.*, 2008). Such conditions often occur in a dense plantation, particularly in maize fields.

Several isoforms of PIFs that promote the SAR are well characterized. PIF Like 1 (PIL1) was the first PIF identified with a direct role in shade avoidance (Salter *et al.*, 2003). Leivar *et al.* (2008) showed that PIF1 plays a central role, while Lorrain *et al.* (2008) found that PIF4 and PIF5 are activated early in the pathway during SAR in Arabidopsis. There are seven PIFs identified in maize, which can be grouped into three phylogenetic clades (ZmPIF3, ZmPIF4, and ZmPIF5) (Wang *et al.*, 2016).

Zhang *et al.* (2023) identified the TF ZmWRKY28 in maize that directly interacts with and activates *ZmPIF4.1.* Among the seven *ZmPIF* genes identified so far, *ZmPIF4.1* showed the strongest effect in restoring SAR symptoms when complemented in Arabidopsis *pif* quadruple mutants (Wu *et al*., 2019). The authors show that, like *ZmPIF4.1*, overexpression of *ZmWRKY28* induces a constitutive SAR phenotype in Arabidopsis including early flowering, elongated petioles, and reduced leaf area. Previously, Sheehan *et al.* (2007) showed similar shade responses in *phyb* mutants in maize. From a differential expression analysis by RNA sequencing, the authors revealed a 50% reduction of *ZmPIF4.1* expression in *zmwrky28* mutants compared with wild-type seedlings. Interestingly, *ZmWRKY28* only affects seedling etiolation but not seed germination, indicating the involvement of other factors in the germination process. Zhang *et al.* (2023) also found a higher expression of *ZmWRKY28* in the stem, mesocotyl, and coleoptiles in light-deprived plants, and the expression decreased upon white light treatment. Seedlings of *zmwrky28* mutants showed a shorter mesocotyl in the dark, while *ZmWRKY28* overexpression lines had a long mesocotyl in the presence of darkness. Protein–protein and protein–DNA interaction assays identified several light-dependent factors including ZmPIF4.1 that interact with ZmWRKY28, and these are regulated sequentially in the SAR induction pathway. These findings open up interesting next steps to investigate whether ZmWRKY28 regulates *COP1*, *HY5*, or other types of *PIF* genes of the light-dependent pathways (Fig. 1).

**Fig. 1.**
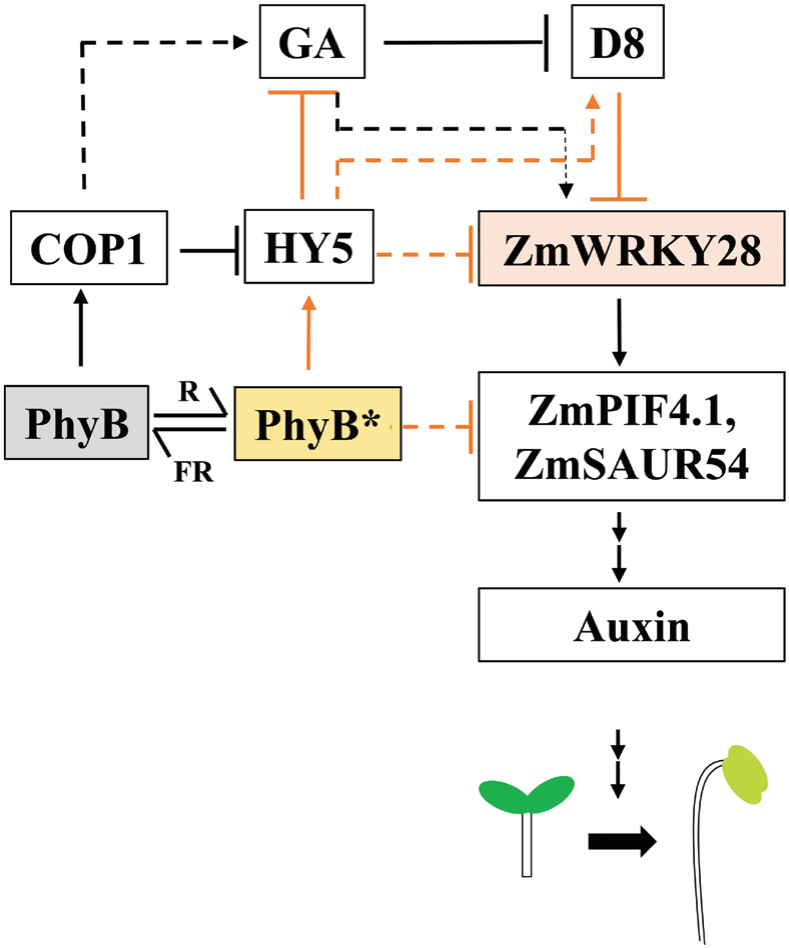
ZmWRKY28 connecting GA–DELLA and COP1–HY5 pathways. PhyB is activated upon sensing the high red (R) to far-red (FR) light ratio. Activated PhyB (PhyB*) blocks COP1 to degrade HY5 and induces photomorphogenesis. HY5 also blocks the GA response, thus keeping DELLA (D8) active. D8 represses ZmWRKY28 and subsequent downstream steps of the skotomophogenic pathway. In the dark, PhyB activates COP1 to degrade HY5. In the absence of Hy5, GA degrades D8. Without the D8 suppression, ZmWRKY28 activates the expression of skotomopogenic pathway genes [*ZmPIF4.1* and *SMALL AUXIN‐UP RNA 54* (*SAUR54*)], and eventually induces other hormones responsible for skotomophogenesis (i.e. auxin). Black and orange arrows indicate dark- and light-activated pathways, respectively. Plain arrows indicate direct and dashed arrows indicate indirect/speculative regulation. This figure shows only the components discussed by Zhang *et al.* (2023).

Given the role it plays in regulating *ZmPIF4.1*, Zhang *et al.* (2023) explored ZmWRKY28 further. Being one of the largest TF families in plants, WRKYs are involved in diverse functions including growth and development, and response to mechanical and biochemical stimuli (Bakshi and Oelmüller, 2014). WRKYs can interact with other TFs, receptors, and kinases, and can participate in dynamic regulatory networks. The DNA- binding domain of WRKY proteins is 60 amino acids long with a conserved WRKYGQK heptapeptide motif at the N-terminus and a zinc-finger-like motif at the C-terminus. Both the motifs of the WRKY domain are responsible for the binding to the TTGAC(C/T) *cis*-element, also known as the W-box, in the promoter region of their target genes. WRKY proteins are classified into three major subgroups (I, II, and III), and these subgroups are further divided into classes with amino acid variations in the conserved domains. Zhang *et al.* (2023) identified that ZmWRKY28 belongs to subgroup IIc and contains a typical WRKYGQK domain and a zinc finger motif (C-X4-C-X23-H-X1-H). WRKY12 and WRKY13 are the closest Arabidopsis homologues of ZmWRKY28. They function in flowering under short-day conditions and are affected by the phytohormone gibberellin (GA). Zhang *et al*. (2023) also found an association of ZmWRKY28 with the the GA–DELLA hormone system. GA–DELLA is one of the key regulatory systems targeted in the ‘green revolution’. GA induces plant growth in the dark by degrading growth repressor proteins of the DELLA family (Alabadí *et al.*, 2004). The revolutionary DELLA gain-of-function mutant in wheat (known as *Reduced height-1*, *Rht-1*) is resistant to GA-mediated degradation and gives the desired dwarf or semi-dwarf plants with a higher harvest index (Pearce *et al.*, 2011). Among 10 maize DELLA genes, the gene *DWARF PLANT8* (*D8*) is an orthologue of wheat *Rht-1* (Harberd and Freeling, 1989; Peng *et al.*, 1999). Zhang *et al.* (2023) showed that D8 is a repressor of *ZmWRKY28*. Transactivation assay showed that the *ZmPIF4.1* activation by ZmWRKY28 is countered by D8, indicating that D8 works upstream of *ZmPIF4.1* transcription. Furthermore, phenotype assessments were performed on seedling development upon treatment with GA and its inhibitor paclobutrazol (PAC) (Zhang *et al*., 2023). A different sensitivity was observed for the wild-type and *zmwrky28* lines. In addition, ZmWRKY28-overexpressing Arabidopsis lines showed reduced sensitivity to PAC treatment. Since *zmwrky28* mutants did not exhibit a complete insensitive phenotype when treated with GA, the authors speculated the possibility of other factors being involved in GA-mediated seedling development. Therefore, it can be postulated that GA is one of the regulators of *ZmWRKY28* activation while D8 reverses the activity (Zhang *et al.*, 2023). In wild-type plants, GA degrades D8 and activates the hormonal signals for stem elongation, associated with skotomorphogenesis (Fig. 1).

Optimum plant density is one of the key factors for achieving maximum yield in maize (Zhang *et al.*, 2021). An individual plant shows maximum productivity in a widely spaced plantation; however, areas of low-density cropping are prone to higher weed growth. On the other hand, under high planting density, plants compete for light, which can in turn reduce crop yield. Some plants have evolved shade tolerance strategies through adapting their physiology, and hence the yield remains unaffected under low-light conditions. Thus, generating shade-tolerant plants is the target for breeders to achieve high-yielding crops in a dense plantation. Being a regulator of the SAR, there is the potential that *ZmWRKY28* loss-of-function lines may also impact maize yield; this deserves further investigation. For example, allele screening for *ZmWRKY28*, in addition to *D8* can be done for identifying lines with the ability to counter the skotomophogenic effect in maize. Furthermore, polymorphisms in the *cis*-regulatory regions of ZmWRKY28-targeted genes can potentially block or weaken the activity of GA signal transmission down the skotomorphogenic pathway. Following this first characterization of ZmWRKY28 by Zhang *et al*. (2023), genome editing to introduce mutations either on the *cis*-regulatory sites for ZmWRKY28 binding or on the conserved domains of ZmWRKY28 could be conducted, followed by phenotype evaluation. Identifying variations in *ZmWRKY28* associated with shade tolerance and/or higher yields can be a future target to determine whether *ZmWRKY28* has breeding potential. Usually, especially in a crop such as maize, breeders have not exploited *ZmWRKY28* as a target. Therefore, this research opens up a new avenue for maize crop improvement.
